# When Celibacy Matters: Incorporating Non-Breeders Improves Demographic Parameter Estimates

**DOI:** 10.1371/journal.pone.0060389

**Published:** 2013-03-29

**Authors:** Deborah Pardo, Henri Weimerskirch, Christophe Barbraud

**Affiliations:** 1 Centre d'Etudes Biologiques de Chizé, Villiers-en-Bois, France; 2 British Antarctic Survey, Cambridge, United Kingdom; University of Plymouth, United Kingdom

## Abstract

In long-lived species only a fraction of a population breeds at a given time. Non-breeders can represent more than half of adult individuals, calling in doubt the relevance of estimating demographic parameters from the sole breeders. Here we demonstrate the importance of considering observable non-breeders to estimate reliable demographic traits: survival, return, breeding, hatching and fledging probabilities. We study the long-lived quasi-biennial breeding wandering albatross (*Diomedea exulans*). In this species, the breeding cycle lasts almost a year and birds that succeed a given year tend to skip the next breeding occasion while birds that fail tend to breed again the following year. Most non-breeders remain unobservable at sea, but still a substantial number of observable non-breeders (ONB) was identified on breeding sites. Using multi-state capture-mark-recapture analyses, we used several measures to compare the performance of demographic estimates between models incorporating or ignoring ONB: bias (difference in mean), precision (difference is standard deviation) and accuracy (both differences in mean and standard deviation). Our results highlight that ignoring ONB leads to bias and loss of accuracy on breeding probability and survival estimates. These effects are even stronger when studied in an age-dependent framework. Biases on breeding probabilities and survival increased with age leading to overestimation of survival at old age and thus actuarial senescence and underestimation of reproductive senescence. We believe our study sheds new light on the difficulties of estimating demographic parameters in species/taxa where a significant part of the population does not breed every year. Taking into account ONB appeared important to improve demographic parameter estimates, models of population dynamics and evolutionary conclusions regarding senescence within and across taxa.

## Introduction

Non-breeding individuals can represent a substantial part of populations mainly as immature but also as breeders skipping reproduction, especially in long lived species. Reproductive skipping is a widespread phenomenon found in many taxa (e.g. [1]–[3]). Skipped breeders, the target of this study, include individuals that do not attempt to breed due for example to a lack of available mates, physiological problems, competitive inferiority, disease, limited experience or genetic quality. Proximate reasons for skipping breeding have been linked in several taxa to body condition which might be environmentally dependent [4], with the necessity to reach certain threshold before engaging in reproduction [3], [5] or having acquired sufficient experience [6]. Skipping can also be the outcome of a breeding strategy by itself such as in biennially breeding species. In such species, skipping is related to the high energetic costs associated to and/or the length of the previous breeding attempt [7], [8]. Despite their numerical importance (skipped breeders can represent more than half of the adult part of the population certain years) non-breeding individuals are most often ignored when studying demographic parameters and modelling population dynamics, mainly because they are more difficult to observe. In population dynamics, abundance and demographic parameters such as adult survival are often estimated from the breeding component of the population since it is the most accessible for monitoring (but see [9]). Nevertheless, skipped breeders can be of considerable importance in population dynamics. For example it was shown that they can act as a buffer in case of high adult mortality and replenish the breeding population avoiding population crashes [10]. Interestingly, part of the individuals skipping breeding can in some cases be observable at breeding sites. One of the reasons of their presence on breeding grounds can be to look for a new partner in case of the loss of the previous one or to look for another nest when breeding failures accumulate. To our knowledge, few studies have been studying the behaviour of these observable non-breeders, and information on their detection at breeding sites has only been rarely used to improve estimates of demographic traits. Skipped-breeders that are not detected at colonies (Unobservable Non-Breeders (UNB)) can be taken into account in capture-mark-recapture models by adding unobservable states [11]. The substantial number of non-breeding individuals that still visit colonies and are detectable (we call them Observable non-breeders ONB) are most of the time ignored in demographic studies on several species [12], [13], [14]. Our aim here is thus to unravel the potential contribution of ONB on the performance of several demographic trait estimates using a complex multi-state model that also incorporates UNB. Survival and breeding success will be studied. Additionally other less studied demographic traits will be incorporated: the probability of returning to the breeding grounds that can only be calculated when ONB are taken into account and of the probability of breeding knowing return.

As a case study, we estimated demographic traits in a long-lived quasi-biennial breeding seabird species, the wandering albatross (*Diomedea exulans*). In such species individuals generally skip the next breeding occasion after a successful breeding event (when they managed to raise their offspring till emancipation). If they fail early in the breeding season, they may attempt to breed again the following year. Most non-breeding individuals are taking a sabbatical year after a successful or late failed breeding event and stay at sea without returning to breeding colonies [11]. As the proportion of ONB may change with age due to life-history trade-offs between survival, current and future reproduction, the performance of estimators between models that consider ONB or not, was assessed under constant (age-independent) and age-dependent variations of the demographic trait investigated.

## Materials and Methods

### Study species and field methodology

Monitoring of wandering albatrosses started in 1960 at Possession Island in the Crozet archipelago, French Southern Territories (46° S; 52° E) and all chicks were systematically ringed since 1966. The breeding cycle of this monogamous species lasts almost a year, therefore few (≈6%) successful pairs (those that fledge a chick) attempt to breed for two consecutive years and only ≈4% succeed, the species is therefore considered as quasi-biennial breeding [11]. The greatest chance of breeding again the following year (≈90%) comes for those pairs that did not attempt to breed (≈100%) or that failed early in the breeding season (during incubation). However, if failure occurs later during the chick growing period only ≈70% of individuals attempt to breed again, the others taking a sabbatical. From mid-November to mid-December pre-breeding adults are checked over the whole island. From mid-January to mid-February at least 3 visits are made every 10 days to obtain the identity of partners and their breeding status, all new individuals are ringed with a uniquely-numbered stainless steel-band. In mid-April, June and August nests are checked and the chick status noted (alive/dead). During all visits, non-breeding individuals are searched for and their identity determined (from ring number) when possible. From mid-September to mid-October fledglings are ringed. Between 1966 and 2010, 8929 chicks were ringed on Possession island.

Based on this monitoring design the breeding status assigned to each individual at the end of the breeding season is the result of all the observations made during the consecutive visits. Therefore we are certain of the status of failed breeders on eggs and chicks and of successful breeders (given their very high detection probability). For assigning the observable non-breeding state, the same field protocol is used, although detection probability is much lower since their identity is much harder to ascertain. Non-breeding individuals present at breeding colonies exhibit a specific behaviour (not tied to a nest, wandering in the colony between nests of conspecifics) that can be easily differentiated from the behaviour of individuals engaged in breeding activities (typical position of incubating birds on their nest, chick feeding). Therefore we are fairly confident with the fact that individuals that were assigned a non-breeding state are indeed non-breeders. Unobservable non-breeders are defined directly through the individual capture histories as a zero the year after a breeding event followed by a return to the breeding colonies. We can be quite confident that this zero reflects that the bird was non-breeder this year because of the very high detection probability of breeders, and that it was not dead because it came back later. However, there is less confidence that an unobservable non-breeder could have come back to the colony, thus being an observable non-breeder. Indeed it could have been missed due to the much lower detection probability of observable non-breeders.

This long-term monitoring program is part of Program IPEV (French Polar Institute) N° 109 lead by H. Weimerskirch. It has received approval from the CNRS Ethics Committee (Comité d'Ethique pour l'Expérimentation Animale du CNRS) n°MP101122105111 and from The Préfet des TAAF (Arrêté N° 2011/95 du préfet des Terres Australes et Antarctiques Françaises), after advice from the CNPN (Comité National de la Protection de la Nature) and from CEP (Comité de l'Environnement Polaire).

### Model building and selection

A reproductive state was assigned to each individual for a given year: failed breeder at the egg/chick stage (FBE or FBC, respectively), successful breeder (SB), or ONB. To model biennial breeding we considered three unobservable states for individuals that skipped breeding according to their previous breeding status (PFB, PSB, PONB, where P = Post; [12], [15]). These three states were pooled together during model selection under the global state of Unobservable Non-Breeders (UNB). To assess the effect of including ONB on demographic parameter estimates, we built two multi-state models. The first one (MSM) did not consider the presence of ONB individuals in the population, whereas the second (MSM_ONB) included it (Fig. S1 and Appendix S1). In model MSM_ONB, 5 key demographic traits were studied: survival, return, breeding, hatching and fledging probabilities, taking into account detection probability. Return probability could not be estimated separately from breeding probability with model MSM since it needs information from ONB individuals (Fig. S1). Therefore the product of return and breeding probabilities estimated from model MSM_ONB was compared to the breeding probability estimated from model MSM.

To achieve an efficient model selection we chose to follow the steps of Grosbois & Tavecchia (2003; [16]): we proceeded to model selection on each trait independently and obtained a composite model that compiled the best model structure for each trait (see Appendix S1 for full details). Model selection was done fully on the most complex life-cycle (MSM_ONB). In order to compare parameter estimates of MSM_ONB with MSM, the age trends and state structure of the MSM_ONB composite model were adapted and applied to the MSM life-cycle (i.e. probability of return removed, probabilities of breeding, hatching and fledging for birds that were ONB the previous year removed, and detection probability of ONB removed). Similarly, to compare age-dependent models with constant models, age-effects were removed from the composite models of MSM_ONB and MSM.

### Measures of estimates' performance

We used three performance measures [17] to compare estimates from both models: bias, precision and accuracy. Because MSM_ONB had more information (136 parameters) than MSM (115 parameters), MSM_ONB was considered as a reference model necessary to compare the three estimates incorporating or not the ONB state. Bias (B) represents the difference in mean estimates between the reference model MSM_ONB (A) and MSM (E): 
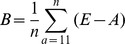
, where *n* is the number of age-classes *a* which is 32 from 11 to 42 years old. Standard deviation (SD) was used as a measure of the precision of estimates to illustrate the variability around mean estimates: 
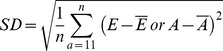
. This formula gives distinct precision measures for models MSM and MSM_ONB.

Root mean squared error (RMSE) measures accuracy. It is a very interesting measurement since it combines both information on bias with the differences in mean of estimates from MSM and MSM_ONB, and precision with the differences in SD of estimates from MSM and MSM_ONB: 
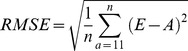
.

These three formulas are given for the age-dependent framework. To apply them in the constant framework, we just have to consider *n* = 1. All performance measures were calculated from ages >10, since most traits were modelled from 10 years old onward, the mean age of recruitment (see Appendix S1, S2 and Table S1). Each measure of performance is calculated from the mean of parameter estimates, yet the SE (Standard Error) of parameter estimates increases with age due to a lower sample size in the oldest age classes. Therefore to include this measure of uncertainty we calculated the SE of each measures of performance based on the delta method (Table 1).

**Table 1:** Summary of averaged performance of estimators across ages [Bias, Standard Deviation (SD), Root Mean Square Error (RMSE) and their respective Standard Errors (SE)], for each demographic trait and corresponding previous state: F = Failed, S = Successful, B = Breeder, E = Egg, C = Chick, NB = Non-Breeder.

	Bias (x100)	SE bias	SD MSM	SE SD MSM	SD MSM_ONB	SE SD MSM_ONB	% SD gained	RMSE	SE RMSE
Survival	0.819	0.004	0.054	0.001	0.045	0.001	17	0.012	0.000
Breeding FBE	−9.712	0.005	0.025	0.004	0.019	0.000	24	0.097	0.000
Breeding FBC	−7.719	0.016	0.113	0.001	0.093	0.001	18	0.084	0.001
Breeding SB	−2.247	0.004	0.055	0.000	0.031	0.000	43	0.035	0.000
Breeding NB	7.385	0.003	0.007	0.000	0.005	0.000	31	0.075	0.000
Hatching FB	0.550	0.008	0.081	0.001	0.077	0.001	4	0.007	0.000
Hatching SB	0.456	0.014	0.126	0.001	0.123	0.001	2	0.006	0.001
Hatching NB	1.054	0.003	0.001	0.000	0.006	0.000	−508	0.013	0.000
Fledging FB	0.072	0.006	0.017	0.001	0.016	0.001	5	0.001	0.000
Fledging FB	0.082	0.012	0.033	0.001	0.032	0.001	4	0.002	0.001
Fledging NB	−0.009	0.005	0.016	0.001	0.016	0.001	3	0.001	0.000
Detection B	9.111	0.013	0.346	0.003	0.387	0.003	−12	0.111	0.000

As opposed to the MSM_ONB capture-recapture model, the information brought by the Observed Non-Breeders is not taken into account in the MSM capture-recapture model. “% SD gained” represents the improvement of SD when the ONB state is considered in model MSM_ONB.

## Results

### Model parameters

Model selection (Table S1 and Appendix S2) highlighted that all life-history traits decreased with age except the breeding probability of individuals that bred the previous year (Fig. S2, Appendix S2). In general, individuals that did not breed the previous year (UNB and ONB) presented the highest demographic parameters (return, breeding, hatching and fledging probabilities, Fig. S2, Table S2 and Appendix S2). Individuals that failed early in the previous breeding season (Failed Breeders on Egg) had higher demographic parameters than individuals that failed late (Failed Breeders on Chicks) or succeeded. The implications of such results from the evolutionary and ecological point of view are discussed deeply in another paper (Pardo et al. in revision). On average, the proportion of non-breeders that were observable represented approximately a third of all non-breeders including skipped breeders.

### Model performance for constant parameters and across ages

**Figure 1:**
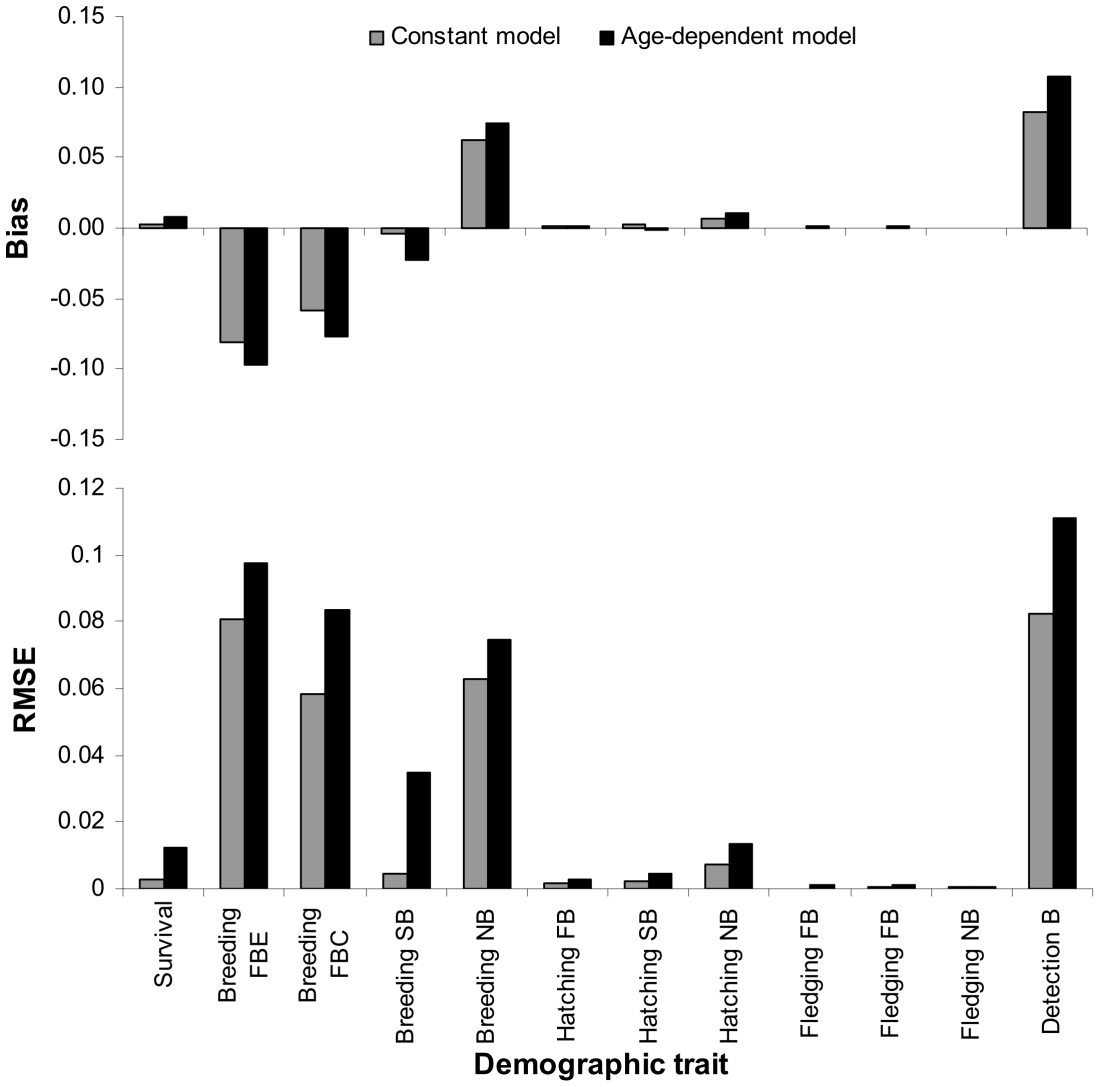
Comparison of bias and RMSE (Root Mean Square Error; ±SE) between constant and averaged age-dependent models on five demographic traits (survival, breeding, hatching, fledging and detection probabilities) taking into account the previous breeding state: F = Failed, S = Successful, B = Breeder, E = Egg, C = Chick, NB = Non-Breeder. As opposed to the MSM_ONB capture-recapture model, the information brought by the Observed Non-Breeders is not taken into account in the MSM capture-recapture model.. The exact same model structure was used for MSM_ONB and MSM capture-recapture models.

Bias between MSM and MSM_ONB was highest for detection probability (9.1%), and for breeding probabilities (between −9.7% and 7.4%; Table 1; Fig. 1). Interestingly the sign of the bias on estimated breeding probability was opposite for individuals that were breeders (FBE, FBC and SB) and non-breeders (ONB) the previous year (Table 1). This suggested contrasted consequences for non-accounting for ONB in multi-state models. The bias on survival, hatching and fledging probabilities was low (less than 1%, Table 1, Fig. 1).

Precision was always better in estimates issued from model MSM_ONB particularly for survival and breeding parameters, except for the hatching success of non-breeders (Table 1). Precision was improved on average by 13% when accounting for ONB, as calculated by averaging the percentage of SD gained in Table 1 while excluding the extreme value of -508% for the hatching probability of non-breeders the previous year,.

The differences in accuracy between MSM and MSM_ONB were the biggest for breeding and detection probabilities (Fig. 1). Although they followed exactly the same patterns, biases were always larger and accuracies lower for age-dependent models than for constant models (Fig 1). This suggests that the influence of ONB individuals on demographic estimates' performance might be even more important when investigating age-effects in populations.

### Age-dependent performance

**Figure 2:**
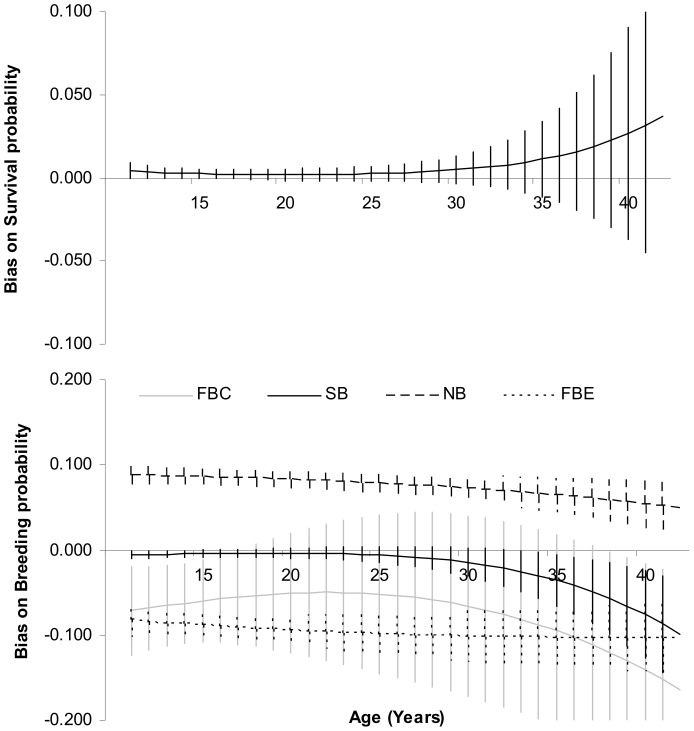
Age-dependent bias differences between models including Observable Non-Breeders or not, for survival and breeding probabilities (±SE) according to four previous breeding states: FBE = Failed Breeder on Egg, FBC = Failed Breeder on Chick, SB = Successful Breeder and NB = Non-Breeders.

Bias on survival probability was positive and increased with age, reaching almost 4% (0.037±0.077) for oldest individuals (Fig. 2). For breeding probabilities, strong age-patterns were also apparent (Fig. 2). Bias for this trait tended to increase with age in absolute value except for birds that were in the unobservable breeding state the year before. The bias became more negative indicating that the breeding probability estimates obtained from model MSM tended to be underestimated compared to those obtained from model MSM_ONB at old ages (Fig. S2 and Appendix 2). It reached substantial values at the oldest ages −0.103±0.041, −0.165±0.130 and −0.099±0.056 respectively for successful breeders, failed breeders on egg and failed breeders on chick the previous year.

## Discussion

This study showed that incorporating information about non-breeding individuals observed at breeding sites in capture-mark-recapture models improved demographic parameter estimates' performance in both constant and age-dependent designs. The influence of accounting for observed non-breeders was greatest when parameters varied by age instead of remaining constant. This suggests that when studying age-effects, available information concerning non-breeders should be taken into account when possible.

We identified several consequences of the decrease in performance when not accounting for observed non-breeders. First, ignoring information brought by observed non-breeders resulted in a relatively large bias in survival. Results suggested that not accounting for observed non-breeders might overestimate the importance of actuarial senescence (senescence on survival probability). This can have strong evolutionary implications for the interpretation of senescence patterns and in comparative studies of senescence across taxa. Additionally, in population dynamics studies, a difference of 4% in survival probability (the value of survival bias at the oldest ages, Fig. 2) in survival can be of considerable importance in long-lived species since the population growth rate is the most sensitive to this trait [18].

Second, ignoring potentially significant variations in return probabilities with age, while ignoring ONB individuals, resulted in overestimating the breeding probability of individuals that bred the previous year, and this bias increased with age (Fig. 2). Breeding probability is a key demographic trait: on the one hand, changes in breeding probability might inform on an individual's condition. At old ages, individuals may reduce their breeding probability as a tactic to reduce breeding costs in long-lived species [19]. Overlooking observed non-breeders may consequently mask senescence patterns. On the other hand, breeding probability can have strong implications in population dynamics as buffering of breeding population size in case of harsh environmental conditions. Improved estimations of this demographic trait are thus of high importance for species' population trends and conservation. Disentangling breeding and return probabilities can bring new insights on the plasticity of demographic strategies [20], [21] or on heterogeneity in individual quality [9], [20], and therefore prove of major importance in the current context of global changes.

Third, missing transitions from the ONB or PONB state to the breeding state resulted in an underestimation of the breeding probability of non-breeders the previous year. Finally, the absence of additional information given by recaptures of non-breeders led to a substantial decrease in detection probability. Such a bias and loss of accuracy can potentially have serious implications on the reliability of other demographic traits since higher detection probability helps stabilizing estimates of transitions between states by improving their accuracy and eventually their identifiability.

We believe that the results of our study are applicable to other species with intermittent breeding. This includes seabirds where non-breeding is frequent, but also other birds, reptiles and mammals. Adding information on observed non-breeders might also help improving demographic estimates in hardly detectable taxa such as turtles, snakes, amphibians, but also in mammals where competitive breeding systems prevent numerous individuals (especially males) from accessing reproduction. More generally, this study highlights that in species where capture/observation of adult individuals is not regular due to intermittent breeding, important biases occur and should be kept in mind when it is not possible to observe at least a few non-breeding individuals as we did in our study. In some cases state assignment might be uncertain or prone to errors. For example, one individual could be classified as a non breeder but may be a failed breeder if breeding and failure went unnoticed, or one individual was observed as a breeder but its state (successful or failed breeders) could not be assigned since it was not observed later during breeding. If state assignment is considered as a serious problem, then it might be useful to use multi-event modelling rather than multi-state modelling, which would allow explicitly modeling and estimating state assignment [22]. A practical consequence of this study is that when designing mark-recapture studies, observations of non breeding animals should be included when possible.

## Supporting Information

Figure S1 **Life cycle graph representing transitions between observable and non-observable states when accounting for observable non-breeders or not.**
(DOC)

Figure S2 **Estimates of age-dependent demographic traits from models incorporating observable non-breeders or not.**
(DOC)

Table S1 **Deviance of several models tested to select the best age and previous breeding state structure on five life-history traits and detection probability of Wandering albatrosses at Crozet Islands from 1966 to 2010.**
(DOC)

Table S2 **Estimates of demographic parameters and detection probability from models incorporating observable non-breeders or not in the constant framework.**
(DOC)

Appendix S1 **Model construction and methodology for model selection.**
(DOC)

Appendix S2 **Results of model selection.**
(DOC)
