# Supplementary material for: When Celibacy Matters: Incorporating Non-Breeders Improves Demographic Parameter Estimates
Source: PLoS One. 2013 Mar 29;8(3):e60389. doi: 10.1371/journal.pone.0060389 (PMC3612038; doi:10.1371/journal.pone.0060389)

S1.1: Life cycle graph representing transitions between observable and non-observable states when accounting for non-breeders (left) or not (right). Both datasets are exactly the same, but in the latter observations of non-breeders are considered as zeros in the capture histories whereas as a state *per se* in the former. The chick stage (C) was added so that age could be calculated directly. Chicks can only pass into breeding states at the minimum age of 5. Observables states (white) include successful breeder (SB), failed breeder on egg (FBE) and failed breeder on chick (FBC) and the observable non-breeding state (ONB). Transitions between observable states are represented by bold double arrows. Transitions between an observable state and its corresponding unobservable state are represented with solid double arrows. The three unobservable states (dark grey) are post-successful breeders (PSB), post-observable non-breeders (PONB) and post-failed breeders (PFB). The transitions from unobservable states to observable states are represented in dashed arrows. The “equations” on top of each state correspond to the probabilities of being in each state, where φ, r, β, ω, γ, ρ represent respectively the survival, return, breeding, hatching, fledging and detection probabilities.


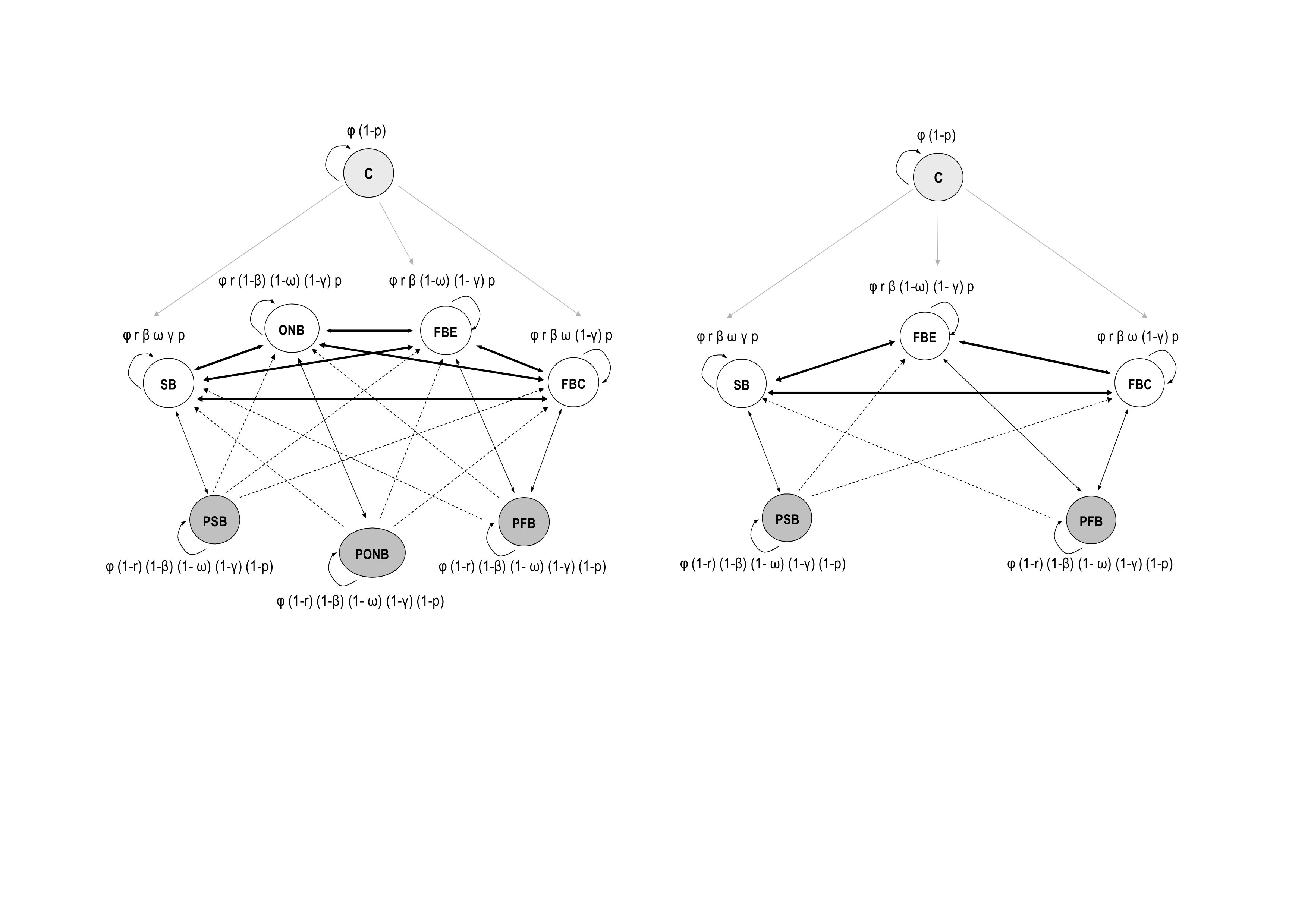

Supplement: Figure S1 — Life cycle graph representing transitions between observable and non-observable states when accounting for observable non-breeders or not. (DOC) [file pone.0060389.s001.doc]
