# Supplementary material for: When Celibacy Matters: Incorporating Non-Breeders Improves Demographic Parameter Estimates
Source: PLoS One. 2013 Mar 29;8(3):e60389. doi: 10.1371/journal.pone.0060389 (PMC3612038; doi:10.1371/journal.pone.0060389)
Supplement: Figure S2 — Estimates of age-dependent demographic traits from models incorporating observable non-breeders or not. (DOC) [file pone.0060389.s002.doc]

Figure S2: Estimates of age-dependent demographic traits from models incorporating Observable Non-Breeders “wONB” in black, or not “nONB” in grey. FBE = failed breeders on egg, FBC = failed breeders on chick, FB = failed breeders on egg or chick, SB = successful breeders, B = breeders, ONB = observable non-breeders, UNB = unobservable non-breeders. Error bars represent standard errors. For readability, the breeding, hatching and fledging probabilities were separated between individuals that bred the year before and individuals that did not bred the year before.
