# Supplementary material for: When Celibacy Matters: Incorporating Non-Breeders Improves Demographic Parameter Estimates
Source: PLoS One. 2013 Mar 29;8(3):e60389. doi: 10.1371/journal.pone.0060389 (PMC3612038; doi:10.1371/journal.pone.0060389)
Supplement: Table S1 — Deviance of several models tested to select the best age and previous breeding state structure on five life-history traits and detection probability of Wandering albatrosses at Crozet Islands from 1966 to 2010. (DOC) [file pone.0060389.s003.doc]

Table S2.1: Deviance of models to select the best age and previous breeding state structure on five life-history traits and detection probability of Wandering albatrosses at Crozet Islands from 1966 to 2010. All models included the state observed non-breeders. Starting from the general model, model selection was done in two steps. In each step, the best model structure retained is in bold. “From” means that the trait varied according to the previous state or the respective pooling of previous states (FBE = failed breeders on egg, FBC = failed breeders on chick, FB = failed breeders on egg or chick, SB = successful breeders, B = breeders, ONB = observable non-breeders, UNB = unobservable non-breeders). “+” and “x” mean that the effect of previous state was additive (same slope) or in interaction (different slopes), respectively. “_” means that the model could not be run due to memory limitations or that it was not necessary to run it. When estimating age-dependent breeding and return probabilities all unobservable states (PFB, PSB and PONB) were pooled in the second step of model selection procedure to improve parameter estimates.

| Model type | Survival | Return | Breeding | Hatching | Fledging | Detection |
| --- | --- | --- | --- | --- | --- | --- |
| **General** | 62987.70 | 62987.70 | 62987.70 | 62987.70 | 62987.70 | 62987.70 |
| Age-effect |  |  |  |  |  |  |
| Linear (+) | 62281.78 | 62046.43 | 61056.15 | 62270.57 | 62223.58 | 62742.24 |
| Quadratic (+) | 61931.04 | 61779.58 | 61062.53 | 62266.44 | 62494.19 | 62167.50 |
| Linear (x) | _ | 61635.68 | **60978.70** | 62266.26 | 62565.37 | 61070.68 |
| Quadratic (x) | _ | 61627.85 | 60982.38 | 62289.77 | 62482.91 | 60962.45 |
| Age-dep. 10 Linear (+) | 62083.27 | 61492.71 | 61073.61 | 62263.12 | 62263.50 | 61244.44 |
| Age-dep. 10 Quadratic (+) | **61764.49** | **61486.51** | 61038.40 | 62263.92 | **61959.73** | 61114.67 |
| Age-dep. 10 Linear (x) | **_** | 64528.55 | 61006.03 | **62260.28** | 62269.73 | 62995.01 |
| Age-dep. 10 Quadratic (x) | _ | 62357.31 | 60999.39 | 62267.21 | 62261.92 | 61975.43 |
| State-effect |  |  |  |  |  |  |
| All pooled | **61764.49** | 63884.39 | 61741.33 | 62434.77 | 62318.21 | 62163.09 |
| Breeding vs Non-breeding | _ | 63006.48 | 61720.60 | 62284.27 | 61966.39 | **60922.66** |
| FB/SB/NB | _ | 61735.56 | 61717.42 | 62266.29 | **61951.03** | 60921.07 |
| FB/SB/ONB/UNB | _ | 61734.18 | 61005.22 | _ | **_** | _ |
| FBE/FBC/SB/ONB/UNB | _ | **61524.79** | **60984.28** | _ | _ | _ |
