# Supplementary material for: When Celibacy Matters: Incorporating Non-Breeders Improves Demographic Parameter Estimates
Source: PLoS One. 2013 Mar 29;8(3):e60389. doi: 10.1371/journal.pone.0060389 (PMC3612038; doi:10.1371/journal.pone.0060389)
Supplement: Table S2 — Estimates of demographic parameters and detection probability from models incorporating observable non-breeders or not in the constant framework. (DOC) [file pone.0060389.s004.doc]

Table S2.2: Estimates of demographic parameters and detection probability from models incorporating observable non-breeders (MSM_ONB) or not (MSM) in the constant framework. FBE = failed breeders on egg, FBC = failed breeders on chick, FB = failed breeders on egg or chick, SB = successful breeders, B = breeders, ONB = observable non-breeders, UNB = unobservable non-breeders.

| Demographic trait | Breeding state | MSM | | MSM_ONB | |
| --- | --- | --- | --- | --- | --- |
|  |  | Estimate | SE | Estimate | SE |
| Survival | All states | 0.944 | 0.002 | 0.947 | 0.002 |
| Return | FBE | _ | _ | 0.978 | 0.011 |
|  | FBC | _ | _ | 0.781 | 0.033 |
|  | SB | _ | _ | 0.289 | 0.015 |
|  | ONB | _ | _ | 1.000 | 0.000 |
|  | UNB | _ | _ | 0.961 | 0.009 |
| Breeding | FBE | 0.882 | 0.011 | 0.820 | **0**.015 |
|  | FBC | 0.558 | 0.027 | 0.640 | 0.036 |
|  | SB | 0.049 | 0.003 | 0.153 | 0.011 |
|  | ONB | _ | _ | 0.460 | 0.011 |
|  | UNB | 0.897 | 0.006 | 0.998 | 0.006 |
| Hatching | FB | 0.780 | 0.010 | 0.782 | 0.010 |
|  | SB | 0.558 | 0.027 | 0.560 | 0.027 |
|  | ONB | _ | _ | 0.832 | 0.012 |
|  | UNB | 0.861 | 0.004 | 0.868 | 0.006 |
| Fledging | FB | 0.948 | 0.006 | 0.948 | 0.006 |
|  | SB | 0.895 | 0.023 | 0.895 | 0.022 |
|  | NB | 0.955 | 0.003 | 0.954 | 0.003 |
| Detection | B | 0.874 | 0.005 | 0.957 | 0.006 |
|  | ONB | _ | _ | 0.221 | 0.012 |
