# Supplementary material for: When Celibacy Matters: Incorporating Non-Breeders Improves Demographic Parameter Estimates
Source: PLoS One. 2013 Mar 29;8(3):e60389. doi: 10.1371/journal.pone.0060389 (PMC3612038; doi:10.1371/journal.pone.0060389)
Supplement: Appendix S1 — Model construction and methodology for model selection. (DOC) [file pone.0060389.s005.doc]

Appendix S1: Model construction and methodology for model selection.

Capture histories were constituted for all individuals ringed as chicks exclusively so that their age was known with precision. Before their first breeding occasion immatures were considered to be absent from the colony (Fig S.1.1). In albatrosses first breeders can have much lower demographic parameters than experienced breeders [1], whatever their age of first breeding (between 5 and 15). They were thus considered separately.

Due to the size of the data set and the complexity of models (9 states, 45 capture occasions, ≈9.000 individuals) the general model was state-dependent, except for survival which was constant. In models with unobservable states not all parameters are identifiable [2]. However as proven by Hunter and Caswell (2009), parameters can all be estimable when breeding and success probabilities are entirely state dependent. Finally for age (a) dependent models, parameters were constrained to be equal at a = 1, a = 2 and a = 3, and a = K-3, a = 2 and a = K-1 (where K is the number of capture occasions; Hunter & Caswell 2009).

We chose to follow the steps of Grosbois & Tavecchia (2003) to achieve an efficient model selection. This approach permitted to solve the technical problems encountered and to compute a minimum number of alternative models. Six series of selection sub-procedures were performed corresponding to one for each life-history trait and detection probability. In each sub-procedure two steps were necessary to get to the best model on a given trait. First we fitted linear and quadratic trends on parameters. Aging theories predict that life-history traits should start to senesce after the age of primiparity [3]. Therefore, models with linear and quadratic trends were also fitted starting at after age 10, the mean and modal age of first breeding [4]. Interactive and additive effects between trends and states were then tested. Secondly we attempted to pool together parameters corresponding to different states to simplify models, and following a rationale based on the hypothesis of differential costs of reproduction according to the previous breeding state. Several gradients of cost of reproduction were tested:

1. Breeders at t-1 versus Non-Breeders at t-1
2. Successful Breeders at t-1 versus Failed Breeders at t-1 versus Non-Breeders at t-1
3. Successful Breeders at t-1 versus Failed Breeders at the Egg stage at t-1 versus Failed Breeders at the Chick stage at t-1 versus Non-Breeders at t-1
4. Successful Breeders at t-1 versus Failed Breeders at the Egg stage at t-1 versus Failed Breeders at the Chick stage at t-1 versus Observable Non-Breeders at t-1 versus Unobservable Non-Breeders at t-1

After the six independent selection sub-procedures, a model referred as the composite model was obtained by combining the total model structure obtained on each trait. It allowed getting parameter estimates that took into account the best selected model structure on each of the five life-history traits together (and detection probability) and therefore gave improved estimates[5].

Goodness-of-fit (GOF) tests were performed for multi-state models [6] using the software U-Care v2.3.2 [7]. Since there is no GOF test for models with unobservable states, we performed the GOF test on the Jolly-Move (JMV) umbrella model with only observable states (SB, FBE, FBC,ONB), excluding the first marking of fledglings at the nest because this state was never re-observed, and thus contributed no information to the test [8]. Model selection was based on Akaike Information Criterion corrected for low sample sizes (AICc). When ΔAICc < 2, the parsimony principle was applied. All models were run in program E-Surge v1.7.1 [9].

References

1. Nevoux M, Weimerskirch H, Barbraud C (2007) Environmental variation and experience-related differences in the demography of the long-lived black-browed albatross. J Anim Ecol 761: 159-167.

2. Hunter CM, Caswell H (2009) Rank and redundancy of multistate mark-recapture models for seabird populations with unobservable states. Modeling demographic processes in marked populations 797-825.

3. Hamilton WD (1966) The moulding of senescence by natural selection. J Theoretical Biology 121: 12-45.

4. Weimerskirch H, Brothers N, Jouventin P (1997) Population dynamics of wandering albatross Diomedea exulans and Amsterdam albatross D. amsterdamensis in the Indian Ocean and their relationships with long-line fisheries: conservation implications. Biological Conservation 79: 257-270.

5. Grosbois V, Tavecchia G (2003) Modeling dispersal with capture-recapture data: disentangling decisions of leaving and settlement. Ecology 845: 1225-1236.

6. Pradel R, Wintrebert CMA, Gimenez O (2003) A proposal for a goodness-of-fit test to the Arnason-Schwarz multisite capture-recapture model. Biometrics 59: 43-53.

7. Choquet R, Lebreton JD, Gimenez O, Reboulet AM, Pradel R (2009) U CARE: Utilities for performing goodness of fit tests and manipulating Capture-REcapture data. Ecography 326: 1071-1074.

8. Crespin L, Harris MP, Lebreton JD, Frederiksen M, Wanless S (2006) Recruitment to a seabird population depends on environmental factors and on population size. J Anim Ecol 751: 228-238.

9. Choquet R, Rouan L, Pradel R (2008) Program E-SURGE: a software application for fitting mulitevent models. Environmental and Ecological Statistics 3: 845-866.
