# Supplementary material for: When Celibacy Matters: Incorporating Non-Breeders Improves Demographic Parameter Estimates
Source: PLoS One. 2013 Mar 29;8(3):e60389. doi: 10.1371/journal.pone.0060389 (PMC3612038; doi:10.1371/journal.pone.0060389)
Supplement: Appendix S2 — Results of model selection. (DOC) [file pone.0060389.s006.doc]

Appendix S2: Results of model selection.

Goodness of fit tests (χ² = 1217.27; d.f. = 1274; p = 0.87) indicated that the general model fitted the data correctly. For simplicity we did not differentiate males and females in the analyses.
